# On the origin of microbial magnetoreception

**DOI:** 10.1093/nsr/nwz065

**Published:** 2019-05-21

**Authors:** Wei Lin, Joseph L Kirschvink, Greig A Paterson, Dennis A Bazylinski, Yongxin Pan

**Affiliations:** 1 Key Laboratory of Earth and Planetary Physics, Institute of Geology and Geophysics, Chinese Academy of Sciences, Beijing 100029, China; 2 Institutions of Earth Science, Chinese Academy of Sciences, Beijing 100029, China; 3 France-China Joint Laboratory for Evolution and Development of Magnetotactic Multicellular Organisms, Chinese Academy of Sciences, Beijing 100029, China; 4 Division of Geological & Planetary Sciences, California Institute of Technology, Pasadena, CA 91125, USA; 5 Earth-Life Science Institute, Tokyo Institute of Technology, Tokyo 152–8551, Japan; 6 Department of Earth, Ocean and Ecological Sciences, University of Liverpool, Liverpool, L69 7ZE, UK; 7 School of Life Sciences, University of Nevada at Las Vegas, Las Vegas, NV 89154-4004, USA; 8 College of Earth and Planetary Sciences, University of Chinese Academy of Sciences, Beijing 100049, China

**Keywords:** magnetoreception, biomineralization, magnetotactic bacteria, exaptation

## Abstract

A broad range of organisms, from prokaryotes to higher animals, have the ability to sense and utilize Earth's geomagnetic field—a behavior known as magnetoreception. Although our knowledge of the physiological mechanisms of magnetoreception has increased substantially over recent decades, the origin of this behavior remains a fundamental question in evolutionary biology. Despite this, there is growing evidence that magnetic iron mineral biosynthesis by prokaryotes may represent the earliest form of biogenic magnetic sensors on Earth. Here, we integrate new data from microbiology, geology and nanotechnology, and propose that initial biomineralization of intracellular iron nanoparticles in early life evolved as a mechanism for mitigating the toxicity of reactive oxygen species (ROS), as ultraviolet radiation and free-iron-generated ROS would have been a major environmental challenge for life on early Earth. This iron-based system could have later been co-opted as a magnetic sensor for magnetoreception in microorganisms, suggesting an origin of microbial magnetoreception as the result of the evolutionary process of exaptation.

## INTRODUCTION

Earth's magnetosphere protects the surface environment from solar wind and cosmic radiation, and has, therefore, been an essential factor in the persistence of life on Earth. It has also provided a natural global positioning system that various organisms have exploited for navigation and migration via the genetically controlled biomineralization of ferrimagnetic iron minerals [1–3]. This iron-based magnetoreception has been identified in microorganisms (prokaryotes and some protists) and diverse animals from fish to mammals, suggesting that it was a primal sensory system of all living systems [4–12]. However, the origin and early evolution of magnetoreception remain major enigmas. It has been proposed that magnetoreception evolved from a pre-existing trait (i.e. biomineralization) through the process of exaptation [13], while, more recently, a non-genetically controlled photoferrotrophy-driven hypothesis has been proposed [14]. How and why biogenic magnetic sensors first evolved remain matters of debate, and resolving these questions is important for understanding the origin and evolution of magnetoreception not only in prokaryotes, but also in eukaryotes. Here, we integrate new data from microbiology, geology and nanotechnology that support an exaptation model for microbial magnetoreception (also known as magnetotaxis) from an initial iron-based system for scavenging intracellular free radicals generated by ultraviolet radiation (UVR) and/or ferrous iron on early Earth.

## THE FIRST MAGNETORECEPTIVE ORGANISMS ON EARTH

**Figure 1.**
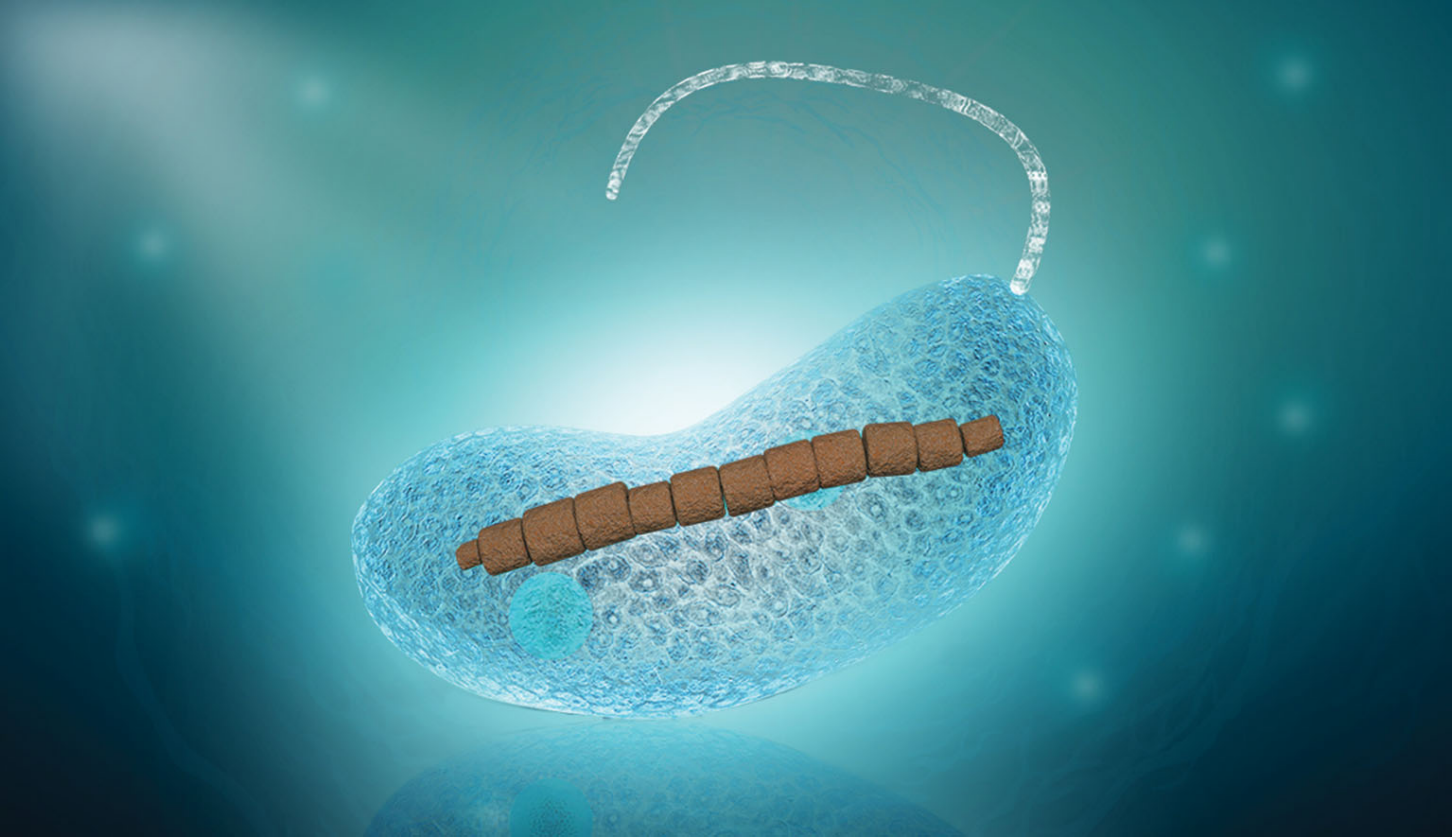
A magnetotactic bacterium (∼2.2 μm in length) with a single chain of Fe_3_O_4_ magnetosomes (brown inclusions). A flagellum is inserted schematically on the right side of the cell. Magnetosomes impart a permanent magnetic dipole moment to the cell and act as an internal compass needle, causing it to align passively along geomagnetic field lines as it swims.

One of the most extensively studied magnetic-sensing organisms are magnetotactic bacteria (MTB)—a group of diverse prokaryotes that synthesize intracellular chain-arranged, nano-sized, membrane-bounded magnetic crystals of magnetite (Fe_3_O_4_) and/or greigite (Fe_3_S_4_) called magnetosomes [2]. Magnetosome chains are the magnetic sensors in MTB, which act as an internal compass needle and cause cells to align passively along the local geomagnetic field (Fig. 1). MTB are the most primitive magnetic-sensing organisms known thus far, with no current evidence of this ability in viruses or the *Archaea*. In addition to the MTB, magnetosome-like structures have been discovered in eukaryotic algae, protozoans and vertebrates [6,7], which led Vali and Kirschvink [15] to propose that the first eukaryotes may have inherited the ability to biomineralize magnetosomes from a magnetotactic alphaproteobacterium during the endosymbiotic development of mitochondria, with subsequent gene transfer to the nucleus.

MTB were discovered independently by Salvatore Bellini and Richard P. Blakemore in 1963 and 1974, respectively [4,16]. These bacteria have a global distribution in aquatic environments from marine to freshwater ecosystems [17]. In addition, they have been shown to be important in the global biogeochemical cycling of Fe as well as other elements, such as S, N, C and P [18–21]. In some environments, magnetosomes from MTB are preserved in sediments or rocks as fossils, referred to as magnetofossils [22,23]. Magnetofossils are important contributions to the remanent magnetization of sediments and have been suggested as biomarkers for reconstructing paleoenvironmental conditions [24]. Magnetofossil records trace an evolutionary history of MTB to the Cretaceous and, with less certainty, to the Precambrian around ∼1.9 Ga [25].

Until a few years ago, all MTB were only assigned to one of two major bacterial phyla: the *Proteobacteria* or the *Nitrospirae* [26]. Use of cultivation-independent approaches (such as 16S rRNA gene-targeting analyses, metagenomics and single-cell genomics) has led to the discovery of previously unidentified MTB lineages, which greatly expands our knowledge of their diversity. MTB have a patchy phylogenetic distribution and are now known to lie within at least five bacterial phyla, including *Proteobacteria*, *Nitrospirae*, *Planctomycetes* and the candidate phyla of *Latescibacteria* and *Omnitrophica*, which suggests that the traits of magnetotaxis and magnetosome biomineralization occur widely in the domain *Bacteria* [17,27–29].

**Figure 2.**
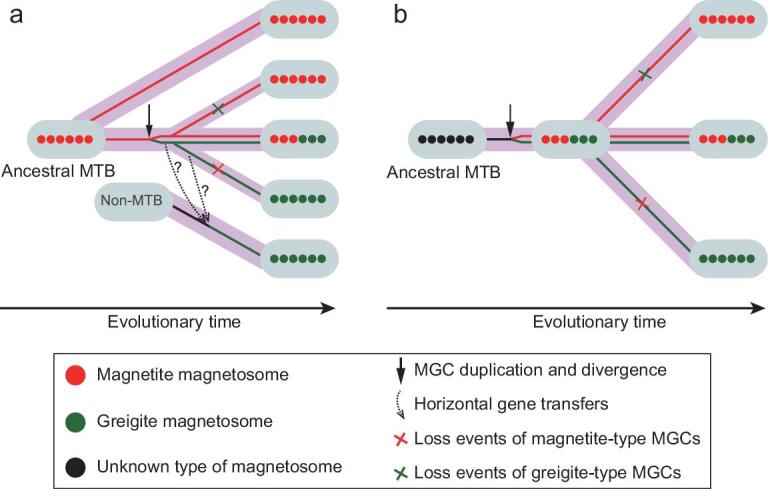
Proposed scenarios for the evolution of magnetotaxis in bacteria at or above the class or phylum taxonomic levels [35]. The last common ancestor of magnetotactic bacteria (MTB) was either (a) magnetite-producing or (b) a bacterium containing an unknown magnetosome type. Both scenarios suggest a monophyletic origin of magnetosome gene clusters (MGCs) from a single common ancestor that existed early in Earth history. Vertical inheritance followed by multiple independent gene losses is a major force that drove the evolution of magnetotaxis in bacteria at or above the class or phylum levels [35,36], while, within lower-level ranks, the evolutionary history of magnetotaxis appears to be much more complicated (e.g. [81–83]).

Molecular, genetic and genomic advances in MTB have led to the identification of a large gene cluster (referred to as a magnetosome gene cluster or MGC) containing a group of genes involved in magnetosome biomineralization and in construction of the magnetosome chain [30–35]. Because of their essential roles in magnetotaxis, comparative and phylogenetic analyses of MGCs from different MTB taxonomies can shed light on the origin and evolution of microbial magnetoreception in bacteria. Recent expansion of MGCs has enabled the reconstruction of the evolutionary history of MTB, which suggests a monophyletic origin of magnetotaxis from a single common ancestor [33,36,37] prior to or near the divergence between the *Nitrospirae* and *Proteobacteria* phyla during the mid-Archean Eon [38] or maybe even earlier, in the last common ancestor of the *Proteobacteria*, *Nitrospirae*, *Omnitrophica*, *Latescibacteria* and *Planctomycetes* phyla (Fig. 2) [35]. Bacterial magnetotaxis, therefore, appears to be a primal physiological process and the first example of magnetoreception and the first example of controlled biomineralization on Earth.

## THE FUNCTION OF MAGNETOSOMES IN EXTANT MTB

Magnetotaxis is clearly the main function of magnetosomes in extant MTB. The presence of these iron nanoparticles imparts a magnetic dipole moment on MTB cells and enables the cells to orient passively, which then allows them to swim actively along the geomagnetic field direction. In general, MTB also appear to have a ‘polarity’—a preference to swim in a particular direction under oxic conditions; that is, they swim to the magnetic north in the northern hemisphere and to the magnetic south in the southern hemisphere [2], although several types of MTB have the opposite polarity in each hemisphere [39,40]. In conjunction with other tactic responses, such as aerotaxis [41], phototaxis [42], chemotaxis [43] or redox taxis [43], magnetotaxis allows MTB to more efficiently locate and maintain positions in their preferred less-oxygenated microhabitats near the oxic-anoxic transition zone in aquatic environments.

**Figure 3.**
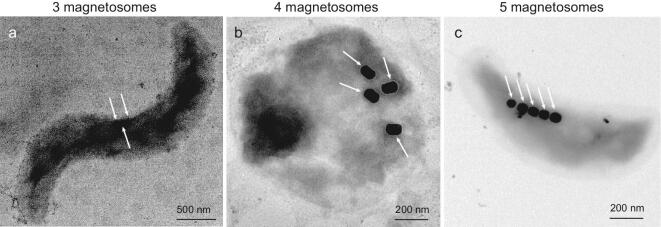
Transmission electron microscope images of uncultured environmental magnetotactic bacteria with (a) three, (b) four and (c) five magnetosome particles per cell (white arrows point to each magnetosome), which indicates that three to five magnetosomes may provide a sufficient magnetic dipole moment for magnetotaxis in these bacteria.

It has been estimated that, for a cell of a *Magnetospirillum* species, a magnetosome chain of 20 Fe_3_O_4_ crystals would provide a sufficient magnetic dipole moment for magnetotaxis [44]. We note, however, that as few as three to five magnetosomes per cell appear to be enough to provide a strong magnetic dipole for orientation in some uncultured environmental MTB (Fig. 3). Some MTB, including ‘*Candidatus* Magnetobacterium bavaricum’ [45] and ‘*Candidatus* Magnetobacterium casensis’ [46] from the *Nitrospirae* phylum, synthesize hundreds of magnetosomes in a single cell—far greater than would be needed for magnetotaxis. The redundancy or ‘overproduction’ of magnetic particles suggests that magnetosomes in MTB may have other functions in addition to magnetotaxis.

Several possible functions have been suggested for magnetosomes, such as iron storage and sequestration, electrochemical batteries, gravity sensors or providing locally strong magnetic fields for enhancing and stabilizing magnetochemical reaction pathways involving free-radical pairs [15,25,47,48]. All of these, however, await confirmation by experimental studies. Recently, however, it has been shown experimentally that Fe_3_O_4_ magnetosomes in some MTB exhibit peroxidase-like activity that can eliminate intracellular levels of reactive oxygen species (ROS) [49]. Moreover, this activity can be further enhanced under irradiation by visible light [50]. These findings indicate strongly the potential functions of magnetosome nanoparticles in the detoxification of ROS or toxic free iron.

## AN ORIGIN OF PROKARYOTIC MAGNETOTAXIS THROUGH EXAPTATION

Exaptation—an evolutionary process by which a biological entity is co-opted for a new role that is unrelated to its initial function [51]—was likely central in the evolution of magnetotaxis. Accumulating evidence indicates that microbial life was present at least since the Archean [52–54] and, as noted above, MTB appear to have originated in the mid-Archean Eon [38]. During the early to late Archean, the primordial atmosphere was anoxic, with *≤*10^−5^ of the present atmospheric level of molecular O_2_ [55,56]. Due to the lack of an effective ozone layer on early Earth, harmful ultraviolet radiation (UVR) was considerably higher than in the present day and would have exerted significant environmental selection pressure on microorganisms in the surface and shallow-water conditions [57]. High UVR levels are detrimental to living microorganisms by either directly causing lesions on native DNA molecules or indirectly through the accumulation of ROS inside cells.

**Figure 4.**
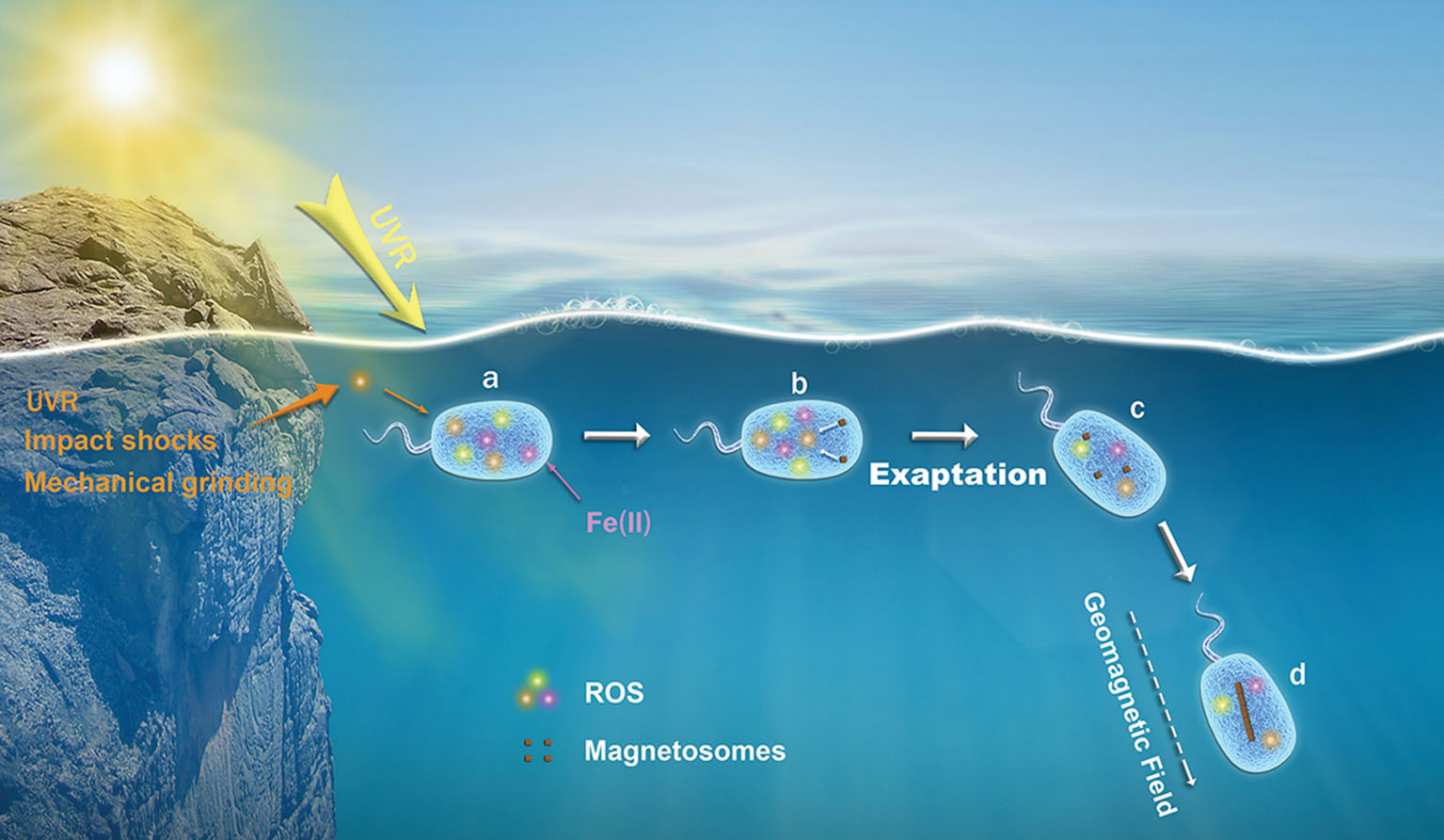
Exaptation model of microbial magnetoreception on early Earth. (a) Reactive oxygen species (ROS) were a major challenge to which ancient life had to adapt. ROS would have been generated and enhanced through ultraviolet radiation (UVR) (yellow), accumulating free Fe(II) inside cells (purple) and/or mineral-induced formation (orange). (b) The ancestral role of intracellular iron-oxide nanoparticles (initial magnetosomes) formed through ancient biomineralization processes was to help early life cope with oxidative stress because of their antioxidant enzyme-like activities and reducing intracellular free iron. (c) Initial magnetosomes were later co-opted to serve an additional new role of magnetoreception as a mineral magnetic sensor. (d) Modification of magnetosomes by natural selection, such as the increase in magnetosome particles and formation of a chain arrangement, would impart a greater magnetic dipole moment to the cell, leading to much more efficient magnetotaxis.

Archean oceans were predominantly anoxic, with abundant dissolved ferrous iron (>30 μm) supplied from mid-ocean ridges, hydrothermal vents and sediment diagenesis [58]. Ferrous iron likely could diffuse passively through the outer membrane of primordial organisms and would have stimulated toxic intracellular ROS levels through the Fenton reaction [59]. Furthermore, ROS might have also been present in aqueous, atmospheric and rock environments on early Earth because of the formation of radical species on mineral surfaces induced by UVR, impact shocks and mechanical grinding [60,61]. ROS accumulation could damage genetic material, deteriorate proteins, cause lipid peroxidation and disturb cellular homeostasis [62]; therefore, dealing with ROS was a major survival challenge for early life on Earth (Fig. 4a).

Extant organisms have evolved various antioxidant systems to detoxify ROS, such as superoxide dismutases, peroxiredoxins and catalases in aerobes and superoxide reductases in anaerobes and microaerophiles [63]. The appearance of appreciable O_2_ concentrations would have led to significant oxidative stress, so it is generally accepted that major antioxidant defense systems evolved prior to the Great Oxygenation Event (GOE), which marked a permanent molecular O_2_ rise in the atmosphere between 2.4 and 2.1 billion years ago [64]. Antioxidant defense systems then radiated massively after the GOE [65]. It remains unclear whether life evolved primordial antioxidant enzymes at or prior to the mid-Archean Eon, although some studies suggest that the last universal common ancestor might have possessed pathways to remove ROS [63,66].

Discovery of intrinsic peroxidase- and catalase-like activities of iron-oxide nanoparticles (IONPs, including Fe_3_O_4_) [67–69] and of peroxidase-like properties of magnetosomes [49,50] leads us to propose that some ancient life forms might have relied on the intracellular biomineralization of IONPs (initial magnetosomes) as antioxidants to cope with ROS stress on early Earth. IONPs have been found to have pH-dependent dual enzyme-like activities in intracellular microenvironments—that is, they catalyse H_2_O_2_ to generate hydroxyl radicals under acidic conditions through peroxidase-like activities and catalyse H_2_O_2_ to H_2_O and O_2_ at neutral and basic pH through catalase-like activities [69]. The median pH of the cytoplasm, periplasm and lumen of the magnetosome vesicle are generally neutral in *Magnetospirillum magneticum* strain AMB-1 cells [70], while the cytoplasmic pH of some uncultured MTB from acidic environments is also close to neutral [29], which indicates that Fe_3_O_4_ magnetosomes may also have catalase-like activity *in vivo*. Compared to traditional antioxidant enzymes, IONPs have enhanced enzyme-like stability under extreme conditions such as a wide range of temperatures (4–90°C) and pH (1–12) [71], which could enable them to maintain antioxidant function in harsh environments.

Microorganisms on early Earth with the ability to mitigate ROS stress would have a competitive advantage. Here, we argue that iron nanoparticle formation (initial magnetosomes) in early primal life had the function of mitigating intracellular ROS toxicity, through their intrinsic antioxidant enzyme-like activities and reducing intracellular toxic free iron (Fig. 4b). With increasing magnetosome numbers, it appears that magnetosomes were co-opted to provide the cell with a magnetic dipole moment for orientation along the geomagnetic field—a formation that was likely established 3–4 billion years ago (Fig. 4c). This primal magnetosensitive structure, which reduces a 3D search to an optimized 1D search along geomagnetic field lines, appears to have further protected ancient life from lethal UVR by allowing efficient directed swimming to deeper water with less O_2_ at or near the oxic-anoxic transition zone either in the water column, the sediment–water interface or deeper in the sediment. For this to occur, natural selection would favor the biomineralization of high-coercivity single-domain magnetic nanoparticles arranged as a chain with dipoles aligned in the same direction to maximize the net magnetic dipole moment for the individual cell to optimize magnetic orientation and navigation (Fig. 4d).

## FUTURE PROSPECTS

An interesting yet unanswered question is: what was the mineral phase of the first magnetic sensor? According to our model, the first magnetosomes should have had antioxidant activities for scavenging intracellular ROS. A growing number of iron nanoparticles, such as Fe_3_O_4_, Fe_2_O_3_ and FeS, have been shown to exhibit enzyme-like activity [72]. It has been suggested that Fe_3_O_4_ might have been the mineral present in the first magnetosomes [37] (Fig. 2a). Alternately, the last common ancestor of MTB could have synthesized an unknown iron-containing biomineral with enzyme-like activity that later, during evolution, perhaps through intracellular changes in enzymatic activity or redox, resulted in the generation of Fe_3_O_4_ and Fe_3_S_4_ particles [35] (Fig. 2b). Identification of this first mineral magnetic sensor remains to be elucidated and is an area of active investigation. The search for putative magnetofossils in older rocks and the reconstruction of ancestral MGC proteins both have the potential to answer this question.

The exaptation model of magnetotaxis imposes an expected evolutionary sequence of magnetosome genes. That is, genes that are involved in magnetosome biosynthesis should have originated earlier than those for magnetosome positioning and crystal size, and for the number of magnetosomes per cell. Genetic studies of MGCs reveal eight (*mamIELMNOBQ*) and six (*mamELMOQB*) magnetosome genes that are essential for Fe_3_O_4_ magnetosome biosynthesis in *Magnetospirillum magneticum* strain AMB-1 and *M*. *gryphiswaldense* strain MSR-1, respectively [31,73]. Homologues of these genes have been identified in MGCs of other MTB, thereby emphasizing their important roles in magnetosome biomineralization. Additional genomic, phylogenetic and evolutionary analyses are clearly necessary to investigate whether these essential genes evolved earlier than those that control magnetosome chain construction (e.g. *mamK* [74] or *mamJ* [75]), magnetosome crystal size (*mms6*, *mmsF*, etc. [31,73]) and the number of magnetosomes per cell. Moreover, studies of the linear organization of magnetosomes and formation of magnetosome membrane vesicles may also shed light on the evolution of the cytoskeleton and vacuole formation in both prokaryotes and eukaryotes [15,76].

It is also clear that further research is required to characterize systematically any additional magnetosome functions beside magnetotaxis in extant MTB. For example, determining whether magnetosome crystals play a role in storing cellular iron, or as an electrochemical battery or gravity sensor, or for promoting magnetochemistry awaits further study. We propose here that Fe_3_O_4_ magnetosome crystals act as a type of iron-oxide nanozyme [69,71] in MTB with neutral intracellular pH by exhibiting catalase-like activity in addition to peroxidase-like activity, although further experimental evidence is required to support this hypothesis. Lastly, why some MTB biomineralize Fe_3_S_4_ magnetosomes as opposed to Fe_3_O_4_ remains unclear, especially considering the generally less perfect chain alignment and poorer crystallinity of Fe_3_S_4_ magnetosomes compared with those of Fe_3_O_4_ magnetosomes [77]. Chemically synthesized Fe_3_S_4_ nanoparticles have also been shown to have peroxidase-like activity [78]. Thus, any further studies, such as those noted above, should also include Fe_3_S_4_-producing MTB.

In space environments, UVR is one of the most significant hazards to living organisms. Therefore, the inferred adaptation of MTB to such high-radiation environments makes them potential model organisms in astrobiology research and may provide an opportunity for studies on the responses of organisms exposed to the near-space and low-Earth-orbit space environments. Such studies could in turn help to better understand the origin and functions of magnetosomes.

MTB are recognized as potentially significant contributors to present-day global iron cycling [19,79]. Recent discovery of an Archean origin of these magnetosensitive microorganisms further suggests that they may have contributed to biogeochemical cycling of iron throughout Earth’s history. We suggest that the ROS-detoxification function of magnetosomes and magnetotaxis capability provided competitive advantages, which might have helped ancient MTB to survive in diverse aquatic environments on early Earth. Considering their uptake of large amounts of environmental iron and intracellular iron biomineralization, MTB likely contributed to iron cycling on early Earth, which further raises the question of whether these microorganisms may have played as-yet-unknown roles in the deposition of banded iron formations that are distributed widely on the remnants of ancient cratons [80]. Future geochemical exploration and magnetic characterization of both extant magnetosomes and magnetofossils will undoubtedly provide new insights into this poorly understood, yet geologically interesting, question.

## CONCLUSIONS

The presence of precise biochemically controlled biomineralization of ferrimagnetic minerals in two domains of life provides strong evidence of Earth's magnetic biosphere. However, the initial origin and subsequent evolutionary history of magnetoreception have not been investigated to any significant degree. We posit that ancient magnetoreception in prokaryotes might have originated via an exaptation process from pre-existing intracellular iron nanoparticles that initially decreased the toxicity of ROS in early life forms. Thus, magnetosome particles in ancient life served a detoxification role and were later co-opted for microbial magnetoreception or magnetotaxis. This exaptation origin of magnetotaxis provides a conceptual model for study of the origin and evolution of magnetoreception, as well as potentially providing a genetic template for other biomineralization systems and mechanisms. With the ever-increasing genomic data from both cultivated and uncultivated MTB as well as advancement of molecular, genetic, chemical and evolutionary technologies, we anticipate great progress in understanding microbial magnetoreception in the near future. Shedding further light on the evolutionary origin of this system will also provide additional constraints on the paleoenvironments under which it evolved as well as on the development of magnetoreception in higher organisms.
